# Evaluation of hand functions and distal radioulnar joint instability in elite wheelchair basketball athletes: a cross-sectional pilot study

**DOI:** 10.1186/s13102-023-00658-8

**Published:** 2023-04-15

**Authors:** Hiroshi Yuine, Hirotaka Mutsuzaki, Yuichi Yoshii, Yukiyo Shimizu, Natsuki Ishida, Taku Yasuda, Koichi Iwai, Kazushi Hotta, Hideki Shiraishi, Kaori Tachibana

**Affiliations:** 1 Department of Occupational Therapy, School of Health Sciences, Ibaraki Prefectural University of Health Sciences, 4669-2 Ami, Ami-Machi, Inashiki-Gun, Ibaraki, 300-0394 Japan; 2 Center for Medical Sciences, Ibaraki Prefectural University of Health Sciences, Ami, Ibaraki 300-0394 Japan; 3 Department of Orthopaedic Surgery, Tokyo Medical University Ibaraki Medical Center, Ami, Ibaraki 300-0395 Japan; 4 Department of Rehabilitation Medicine, Faculty of Medicine, University of Tsukuba, Tsukuba, Ibaraki 305-8575 Japan; 5 Geriatric Health Services Facility Nadeshiko, Tsukuba, Ibaraki 300-4245 Japan; 6 Center for Humanities and Sciences, Ibaraki Prefectural University of Health Sciences, Ami, Ibaraki 300-0394 Japan; 7 Department of Physical Therapy, School of Health Sciences, Ibaraki Prefectural University of Health Sciences, Ami, Ibaraki 300-0394 Japan

**Keywords:** Wheelchair basketball, Distal radioulnar joint instability, Hand function, Ultrasonography

## Abstract

**Background:**

Wrist injury affects wheelchair basketball players’ performance; however, the relationship between distal radioulnar joint (DRUJ) instability and hand functions is unclear. This cross-sectional pilot study investigated DRUJ instability in elite female wheelchair basketball athletes using force-monitor ultrasonography.

**Methods:**

Nine elite female wheelchair basketball athletes (18 wrists) were included in the study. A triangular fibrocartilage complex (TFCC) injury was confirmed using magnetic resonance imaging (MRI). Hand functions were evaluated based on the range of motion (ROM) of wrist palmar flexion, dorsiflexion, radial deviation, and ulnar deviation; grip strength; arm circumference; forearm circumference; and DRUJ instability or pain using the ballottement test. The Mann–Whitney *U* test was used to compare parameters between the TFCC-injured and intact wrists. Radioulnar displacement was measured using force-monitor ultrasonography and pressure data, and the displacement-to-force ratio was used as an indicator of DRUJ instability. The correlation between the DRUJ displacement-to-force ratio and each hand function assessment was evaluated using Pearson correlation coefficient for the TFCC-injured and intact wrists. A generalized linear mixed model (GLMM) was used to estimate the relationship between hand functions and DRUJ instability.

**Results:**

TFCC injuries in seven wrists were confirmed using MRI findings (38.9%). The ulnar deviation ROM values of the TFCC-injured wrist (n = 7) and intact (n = 11) groups were 38.6 ± 8.0° and 48.6 ± 7.8°, respectively. The ulnar deviation ROM was significantly smaller in the TFCC-injured wrists (p = 0.02, r = − 0.54). In the TFCC-injured wrists, no correlation was observed between the displacement-to-force ratio and the hand function assessment. In contrast, the displacement-to-force ratio negatively correlated with grip strength, arm circumference, and forearm circumference in the intact wrists (Pearson correlation coefficient r = − 0.78, − 0.61, and − 0.74, respectively). The GLMM showed that the displacement-to-force ratio significantly affected grip strength, arm circumference, and forearm circumference in the intact group.

**Conclusions:**

In intact wrists, correlations were observed between hand functions such as upper arm/forearm strength and DRUJ stability evaluated using ultrasound. Maintaining and strengthening grip strength, forearm circumference, and arm circumference are associated with DRUJ stability and may be related to TFCC injury prevention in wheelchair basketball athletes.

*Trial registration*: The protocol was registered with the UMIN Clinical Trials Registry (UMIN000043343) [Date of first registration: 16/02/2021].

## Background

Wheelchair basketball, a popular parasport played by approximately 100,000 individuals worldwide [1], requires high levels of performance of the upper limbs, which players use to dribble, pass, and shoot the ball, all while controlling their wheelchairs. It is also a sport that involves a considerable amount of contact because players must defend against opponents by deftly controlling their bodies and wheelchairs. Wheelchair basketball athletes often experience orthopedic diseases of the shoulders, elbows, and hands due to excessive stress placed on the upper limbs from attempting to achieve high levels of performance. Repeated shooting, operating the wheelchair with high acceleration and deceleration or sudden or forceful turning and braking, playing defense and offense while holding the hand rim, and placing hands on the ground when falling may cause upper limb injuries. Wheelchair users who frequently drive wheelchairs with wrist ulnar flexion or perform push-up movements are prone to triangular fibrocartilage complex (TFCC) injuries [2]. One common injury that cannot be overlooked in wheelchair basketball athletes is the TFCC injury [3, 4]. Distal radioulnar joint (DRUJ) instability is associated with the TFCC injury and leads to upper limb functional disorders, such as pain in the ulnar side of the wrist and reduced strength of the forearm’s supinator and pronator muscles [5]. Wrist supporters are used to treat DRUJ instability; however, for wheelchair basketball athletes, rest and fixation using a supporter may reduce performance in competition. As DRUJ instability may affect an athlete’s career, preventing these injuries and reducing the risk of recurrence are important issues.

Assessment of DRUJ instability is essential to investigate the effectiveness of preventive measures and interventions for hand dysfunction. The ballottement test [6] and piano key test [7] are widely used manual methods to clinically evaluate DRUJ instability. These assessments are based on the subjectivity of the examiner, while how DRUJ stability changes using manual stress tests remains nebulous. Furthermore, since DRUJ instability does not always accompany TFCC injuries, quantitative assessments are needed to assess the severity of these conditions. Conventionally, several imaging modalities have been used to evaluate DRUJ instability quantitatively, including a simple method using an ultrasound device [8–11]. This method could be used to evaluate wheelchair users quickly and frequently. In this method, the ultrasonic transducer is attached to a cyclic compression apparatus, and the movement of the radius and ulna bones during compression can be monitored and pressure data can be recorded [11].

The relationships between clinical hand functions and DRUJ instability in wheelchair basketball athletes are poorly understood. We hypothesize that quantifying DRUJ instability in elite female wheelchair basketball athletes could help to clarify the relationships between hand functions and DRUJ instability. The objective of this study was to evaluate the relationships between ultrasound assessments of DRUJ instability and clinical hand functions in elite female wheelchair basketball athletes.

## Methods

### Study design

This study was a cross-sectional pilot study.

### Ethics statements

This study was approved by the Institutional Review Board of Ibaraki Prefectural University of Health Sciences (e306), and the protocol was registered with the UMIN Clinical Trials Registry (UMIN000043343) [Date of first registration: 16/02/2021]. The study was explained to all the participants in writing, and their written informed consent was obtained.

### Participants

Participants were recruited through a poster displayed in the hospital and by explaining the study during a medical check-up for the candidates of the national team. At the medical check-up, which was conducted once a year, the participants joined this study for one day in February of 2021; their medical records were reviewed to collect data regarding underlying diseases, age, dominant hand, competition history, classification points, occupation, and magnetic resonance imaging (MRI) findings. MRI findings confirmed the existence of the injury from the record taken before. The presence of wrist pain was confirmed by inquiry and manual tests. In this study, a TFCC injury was defined based only on MRI findings of the injury, since the assessment timing between MRI and current wrist pain was different. Participants’ sport classification points were determined based on the “volume of action” related to core functions from the International Wheelchair Basketball Federation [12]. All participants were evaluated for DRUJ instability and hand functions. Participants received a summary of assessment results after this research was conducted.

### Evaluation method

Participants were evaluated for DRUJ instability or pain using the ballottement test, followed by radioulnar displacement using force-monitor ultrasonography and determination of range of motion (ROM) of wrist palmar flexion, dorsiflexion, radial deviation, ulnar deviation, grip strength, arm circumference, and forearm circumference.

### Evaluation of DRUJ instability

**Fig. 1:**
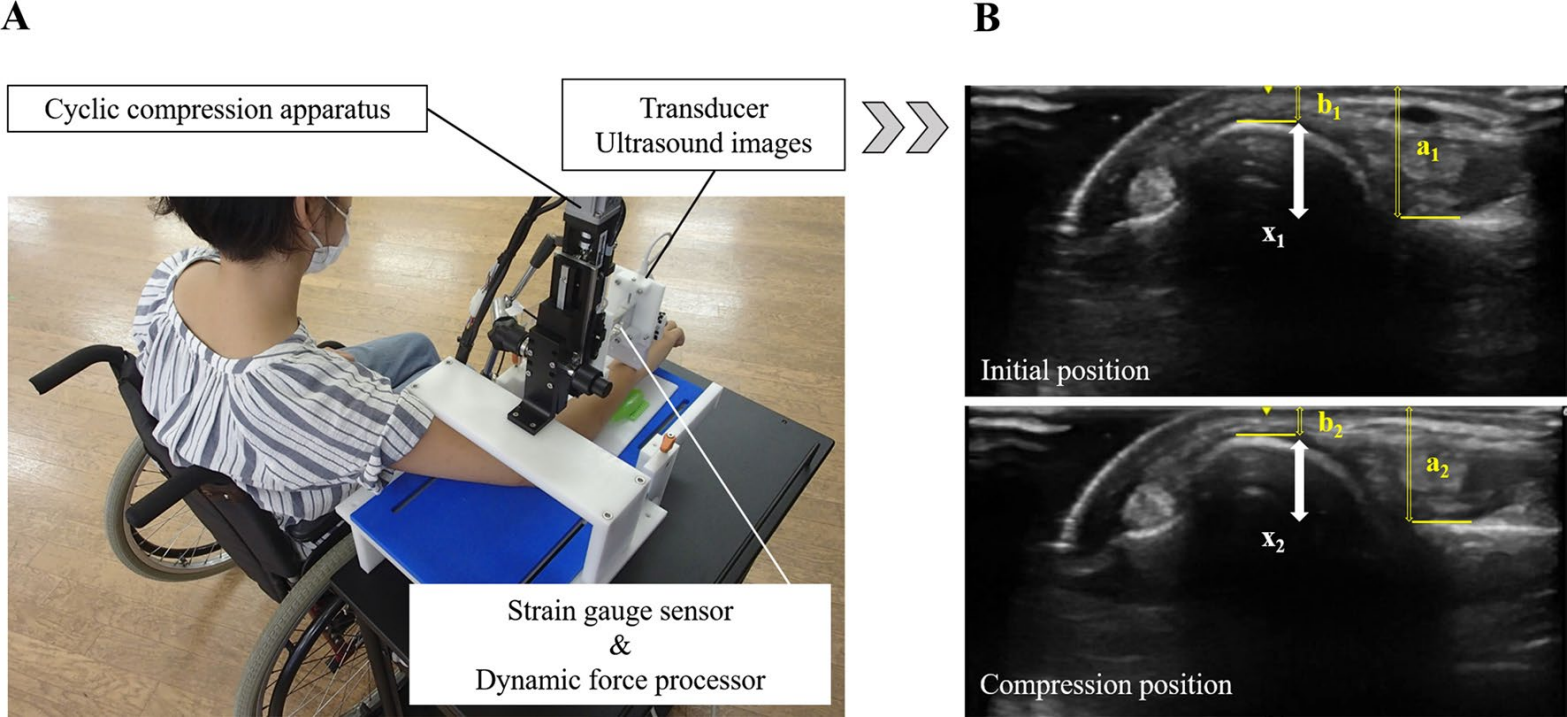
Evaluation of the distal radioulnar joint instability. **A** Force-monitor ultrasonography. **B** Sample of ultrasonographic images

Force-monitor ultrasonography [11] was used to assess DRUJ instability (Fig. 1). This evaluation method uses the transducer of an ultrasound device (LOGIQ e Premium; GE Healthcare, Chicago, IL) attached to a cyclic compression apparatus [13]. The apparatus was portable, and data were collected in a room at the hospital. While applying compression in the palmar direction to the ulnar head with the ultrasonic transducer, the dynamic behavior of the DRUJ under external force was recorded. A pressure force sensor (FS10A; Unipulse, Tokyo, Japan) incorporated into the cyclic compression apparatus was used simultaneously to record the force applied to the wrist. A linear array transducer and ultrasound gel were used for the imaging.

Previous studies were referenced for the measurement procedure [11, 14]. The accuracy of the evaluations with this method was confirmed in a comparison of healthy individuals and those with TFCC injuries accompanied by DRUJ instability [15]. The device settings for high inter-rater reliability were as follows [14]. The transducer moved 3.0 mm in the superior-inferior direction, using a compression cycle of 1.5 Hz.

The measurement procedure for participants was as follows: The participants placed their forearms horizontally on the device platform and sat still with their shoulder abducted, elbow flexed, and forearm pronated. Support was placed beneath the forearm to maintain a limb position to allow effective compression to the ulnar head in the palmar direction. The position of the platform was adjusted to determine the appropriate measurement position. The position was set as follows. First, a cross-section of the DRUJ was depicted on the screen of the ultrasound device, and the long axis of the forearm was fixed in a position where the diameter of the distal ulna was at its largest. Second, the forearm was fixed in a direction perpendicular to the long axis where the center of the distal ulna was in the middle of the ultrasound screen. Finally, the ultrasound transducer was placed against the dorsal side of the ulna as the starting position. The pressure data were reset to 0 in the starting position, and cyclic compression was applied with the transducer on the dorsal surface of the ulnar head in the palmar direction.

The procedure for analyzing the measured ultrasonographic images and pressure data was as follows. For the ultrasonographic images and pressure data, the mean of five compression cycles was used in the analysis. The ultrasonographic images were uploaded into ImageJ software (National Institutes of Health, Bethesda, MD) to extract cross-sectional images of five cycles of compression and non-compression. The distance from the transducer’s surface to the dorsal edge of the radius was defined as “a,” and the distance from the transducer’s surface to the dorsal edge of the ulna was defined as “b.” The radioulnar distance (x) during non-compression and compression was calculated as follows: x = a – b. The radioulnar displacement (X, mm) during non-compression (x_1_) and compression (x_2_) was calculated as follows: X = x_1_ – x_2_. The pressure data recorded the cyclic force applied to the wrist at the start and during compression with a compression force sensor at a sampling rate of 200 times/s. The compression force (N) used in the analysis was the mean amplitude of five compression cycles. The displacement-to-force ratio (mm/N) was obtained using the mean displacement and compression force values.

### Assessment of hand functions

Grip strength was measured twice on each side using a grip strength meter (Takei Electric Industries, Tokyo, Japan), and the mean value was used in the analysis. ROM was calculated for flexion, extension, radial deviation, and ulnar deviation on the left and right sides using a goniometer (GS-100; OG Wellness, Tokyo, Japan). Circumferences of the left and right upper arms and forearms were measured at the maximum level using a tape measure (GS11-004, OG Wellness). Interviews using Quick Disability of the Arm, Shoulder, and Hand (DASH) were conducted to record participants’ daily activities, work, and any difficulties or pain experienced during sports [16]. The validity of the Quick DASH questionnaire has been verified [16]. DRUJ instability was manually evaluated by one physician with the ballottement test for the instability and pain. The ballottement test has been reported to have the highest reliability in manual assessment of DRUJ instability [17, 18].

One occupational therapist who assessed DRUJ instability assessed ROM, and another occupational therapist assessed muscle strength and circumference.

### Statistical analysis

Based on the mean and standard deviation values from previous studies [15], a power analysis was performed with a confidence level of 95% (*α* = 0.05) and a power (1-β) of 80%. Power analyses were performed using G*Power v3.1.9.6 (Faul, Erdfelder, Lang, & Buchner, Germany) software, which estimated a minimal sample size of 14 wrist joints in each group.

MRI findings were used to divide the participants into groups: with and without TFCC injuries. All variables were evaluated for normality in each group using the Shapiro–Wilk test. The Mann–Whitney U test was used to compare data between the two groups. Effect size r was calculated with standard units (Z score) and sample size (n) using the following equation:

$${\text{effect size r }} = {\text{ Z score }}/\surd {\text{n}}$$

Pearson correlation coefficients were calculated with respect to hand functions for radioulnar displacement, applied force, and the displacement-to-force ratio in the TFCC-injured and intact wrists. *r* values (effect size and Pearson correlation coefficient) were interpreted as follows: r value > 0.20 indicated small, > 0.30 medium, and > 0.50 large [19]. Furthermore, a generalized linear mixed model (GLMM) was used to estimate the relationship between hand functions and DRUJ instability. Grip strength; ROM of palmar flexion, dorsiflexion, and radial and ulnar deviation ROM; arm circumference; and forearm circumference were defined as outcome variables. The displacement-to-force ratio, age, and classification were defined as fixed effects. SPSS Statistics version 27 (IBM, Armonk, NY) was used to perform all statistical analyses. P-values < 0.05 indicated statistical significance.

## Results

A total of nine elite female wheelchair basketball athletes (18 wrists of the dominant and non-dominant hands), who were candidates for the national team, were enrolled in this study. The Shapiro–Wilk test showed normal distributions for most datasets, with only the dorsiflexion ROM and arm circumference in the intact wrist group showing a non-normal distribution. Table 1 presents the participants’ characteristics. All participants had been practicing daily and attending training camps for national team candidates. All participants had MRI findings of the presence or absence of TFCC injuries in the last 2.5 years. The TFCC injuries were identified on bilateral wrists of three participants (six wrists) and on the unilateral wrist of one participant (one wrist). In one case of a unilateral injury, the ballottement test showed positive results for both wrists, and the displacement on both sides was 0.96 mm in the assessment using ultrasonography. Table 2 summarizes the results of DRUJ instability and hand function tests in the TFCC-injured and intact wrists. The ulnar deviation ROM was significantly more restricted in the TFCC-injured wrists than in the intact wrists.

**Table 1:** Participant characteristics

Characteristic	Value
Age (years): Mean ± SD	32.1 ± 6.2
Range (min–max)	24–44
Underlying disease: type (n)	Spinal cord disease (4)
	Orthopedic and other diseases (5)
Dominant side:	Right side (9)
Competition history (years): Mean ± SD	12.3 ± 4.4
Range (min–max)	7–21
Classification (points): Mean ± SD	2.83 ± 1.5
Range (min–max)	1–4.5
MRI finding (n):	TFCC-injured wrists (7)
	Intact wrists (11)
Ballottement test: wrist (n)	DRUJ instability (4)
Wrist pain: wrist (n)	4
QuickDASH (points): Mean ± SD	7.58 ± 8.1
Range (min–max)	0–22.7
QuickDASH work score (points): Mean ± SD	5.56 ± 7.9
Range (min–max)	0–18.8
QuickDASH sports score (points): Mean ± SD	13.9 ± 12.8
Range (min–max)	0–31.3

*SD* Standard deviation, *min* Minimum, *max* Maximum *MRI* Magnetic resonance imaging, *TFCC* Triangular fibrocartilage complex, *DRUJ* Distal radioulnar joint, *DASH* Disability of the Arm, Shoulder, and Hand

**Table 2:** Comparison of DRUJ instability and hand functions between the study groups

Characteristic	TFCC-injured wrists (n = 7)	Intact wrists (n = 11)	*p*-value	Effect size r
Displacement (mm): Median (IQR)	0.86 (0.16–0.96)	0.50 (0.27–0.67)	0.29	− 0.26
Mean ± SD	0.67 ± 0.41	0.51 ± 0.24		(small)
Range, min–max	0.06–1.14	0.18–0.96		
Applied force (N): Median (IQR)	4.47 (4.28–4.78)	4.36 (3.39–4.46)	0.13	− 0.37
Mean ± SD	4.53 ± 0.28	3.92 ± 0.96		(medium)
Range, min–max	4.20–5.01	2.17–5.15		
Displacement-to-force ratio (mm/N): Median (IQR)	0.19 (0.04–0.22)	0.12 (0.09–0.18)	0.43	− 0.20
Mean ± SD	0.15 ± 0.09	0.13 ± 0.05		(small)
Range, min–max	0.01–0.24	0.05–0.20		
Grip strength (N): Median (IQR)	309.9 (235.8–362.8)	323.6 (281.9–339.8)	1.0	− 0.01
Mean ± SD	303.9 ± 65.6	305.8 ± 54.7		(none)
Range, min–max	203.5–379.0	209.9–383.0		
ROM, palmar flexion (°): Median (IQR)	75 (60–90)	75 (70–80)	0.86	− 0.04
Mean ± SD	72.1 ± 16.0	74.5 ± 6.9		(none)
Range, min–max	45–90	65–85		
ROM, dorsiflexion (°): Median (IQR)	75 (70–85)	80 (80–85)	0.33	− 0.25
Mean ± SD	76.4 ± 7.5	79.5 ± 6.9		(small)
Range, min–max	65–85	60–85		
ROM, radial deviation (°): Median (IQR)	25 (20–25)	30 (25–35)	0.10	− 0.41
Mean ± SD	23.6 ± 3.8	28.6 ± 6.4		(medium)
Range, min–max	20–30	20–40		
ROM, ulnar deviation (°): Median (IQR)	35 (30–45)	50 (40–55)	0.02^*^	− 0.54
Mean ± SD	38.6 ± 8.0	48.6 ± 7.8		(large)
Range, min–max	30–50	35–60		
Arm circumference (cm): Median (IQR)	29.0 (27.7–29.5)	28.5 (28.2–29.5)	0.66	− 0.11
Mean ± SD	28.5 ± 1.1	29.2 ± 1.8		(small)
Range, min–max	26.7–29.6	27.0–33.0		
Forearm circumference (cm): Median (IQR)	23.5 (22.7–24.0)	24.0 (24.0–24.8)	0.07	− 0.43
Mean ± SD	23.4 ± 0.7	24.5 ± 1.5		(medium)
Range, min–max	22.4–24.6	22.6–27.2		

*SD* Standard deviation, *IQR* Interquartile range, *min, minimum* max, maximum, *DRUJ* Distal radioulnar joint, *ROM* Range of motion, *TFCC* Triangular fibrocartilage complex. *p < 0.05

**Fig. 2:**
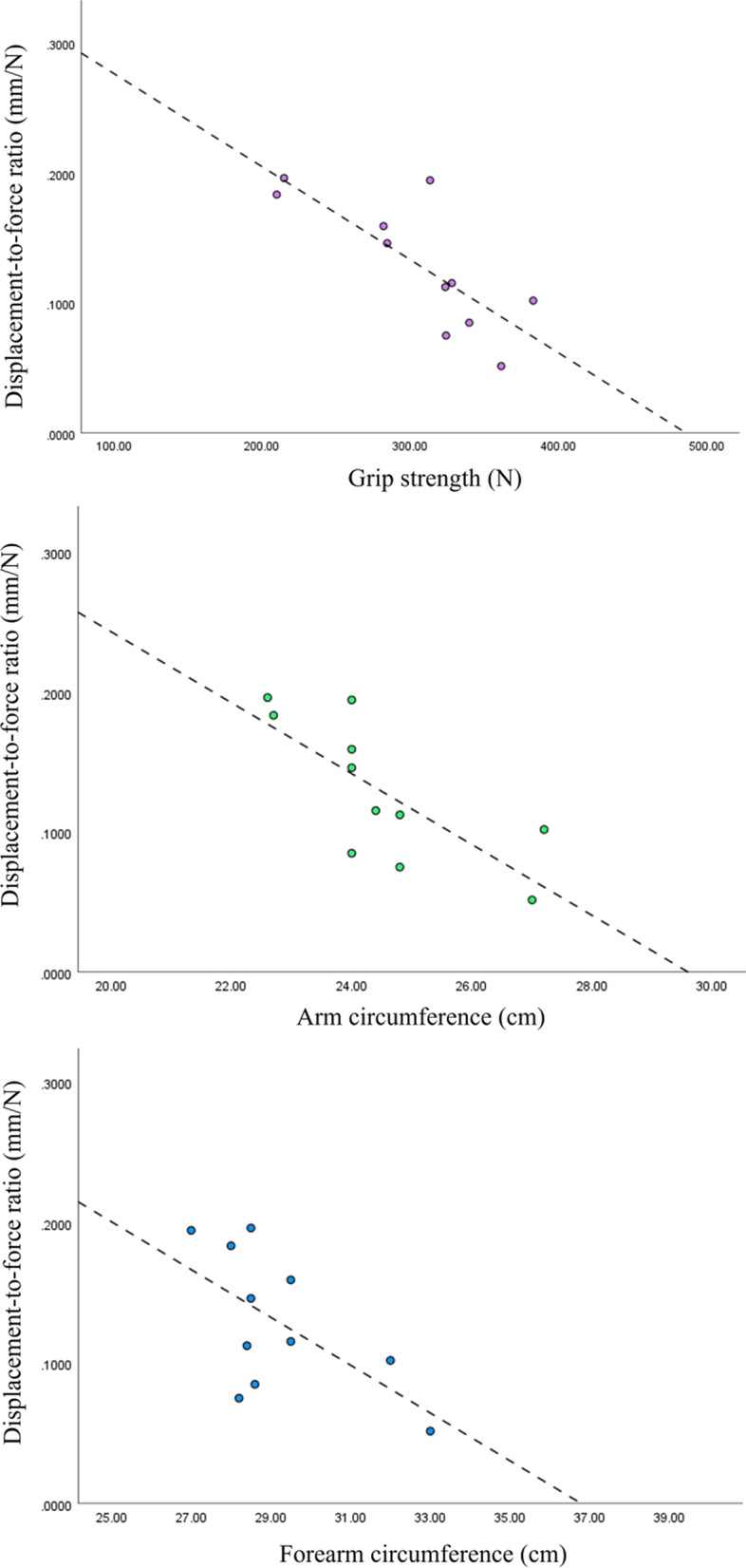
Correlations between distal radioulnar joint instability and hand functions in intact wrists

Correlations were found between DRUJ instability and hand functions in the TFCC-injured and intact wrists (Tables 3 and 4), but no significant correlations were found between DRUJ instability and hand functions in the TFCC-injured wrists. In the intact wrists, there were negative correlations between the displacement-to-force ratio and grip strength, upper arm circumference, and forearm circumference (Fig. 2). The effects of hand functions on DRUJ instability in the intact and TFCC injury groups with GLMM are presented in Table 5. In the intact wrist group, the corrected Akaike’s information criterion (AICc) and Bayesian information criterion (BIC) of Model 2 had the smallest values among those of the three models, while the goodness of fit of the model was the best. In the intact wrists, the displacement-to-force ratio affected grip strength, arm circumference, and forearm circumference. Conversely, in the TFCC injury group, the model with the lowest AICc and BIC was varied based on the outcome measures. In the TFCC-injured wrists, the displacement-to-force ratio affected the grip strength.

**Table 3:** Correlations between DRUJ instability and hand functions in TFCC-injured wrists

Characteristic	Displacement	Applied force	Displacement-to-force ratio
Grip strength: r	0.73	0.35	0.72
*p-*value	0.064	0.44	0.067
ROM, palmar flexion: r	0.06	−0.28	0.09
*p-*value	0.89	0.55	0.86
ROM, dorsiflexion: r	0.60	− 0.44	0.65
*p-*value	0.15	0.32	0.11
ROM, radial deviation: r	0.27	0.70	0.19
*p-*value	0.56	0.079	0.69
ROM, ulnar deviation: r	0.72	− 0.095	0.75
*p-*value	0.066	0.84	0.054
Arm circumference: r	− 0.24	0.031	− 0.24
*p-*value	0.60	0.95	0.61
Forearm circumference: r	0.38	0.12	0.39
*p-*value	0.40	0.79	0.39

*r* Pearson correlation coefficient, *DRUJ* Distal radioulnar joint, *TFCC* Triangular fibrocartilage complex; ROM, range of motion

**Table 4:** Correlations between DRUJ instability and hand functions in intact wrists

Characteristic	Displacement	Applied force	Displacement-to-force ratio
Grip strength: r	− 0.501	0.22	− 0.78
*p-*value	0.12	0.52	0.004^**^
ROM, palmar flexion: r	− 0.14	0.42	− 0.41
*p-*value	0.69	0.20	0.21
ROM, dorsiflexion: r	0.044	− 0.23	0.19
*p-*value	0.90	0.49	0.57
ROM, radial deviation: r	0.058	− 0.29	0.14
*p-*value	0.87	0.38	0.69
ROM, ulnar deviation: r	− 0.24	− 0.59	0.011
*p-*value	0.49	0.058	0.97
Arm circumference: r	− 0.44	0.39	− 0.61
*p-*value	0.18	0.24	0.048^*^
Forearm circumference: r	− 0.45	0.42	− 0.74
*p-*value	0.16	0.20	0.009^**^

*R* Pearson correlation coefficient, *DRUJ* Distal radioulnar joint, *TFCC* Triangular fibrocartilage complex, *ROM* Range of motion. *p < 0.05, **p < 0.01

**Table 5:** Relationship between hand functions and DRUJ instability determined using GLMM

Characteristic	Intact wrists (n = 11)						TFCC-injured wrists (n = 7)					
	Model 1		Model 2		Model 3		Model 1		Model 2		Model 3	
	t	*p*-value	t	*p*-value	*t*	*p*-value	t	*p*-value	t	*p*-value	t	*p*-value
Grip strength												
	2.94	0.019	1.28	0.243	13.2	0.000	− 1.09	0.339	− 1.12	0.346	2.25	0.088
DF ratio	− 4.70	0.002^**^	− 4.38	0.003^**^	− 4.62	0.002^**^	5.23	0.006^**^	4.25	0.024^*^	1.79	0.147
Age	2.18	0.061	0.39	0.709			3.41	0.027^*^	2.95	0.060		
Classification			0.25	0.810	2.15	0.064			− 0.63	0.574	0.74	0.503
AICc	83.3	75.6	82.0	45.9	40.0	47.3						
BIC	82.7	74.7	81.4	43.3	35.1	44.7						
ROM, palmar flexion												
	3.30	0.011	1.01	0.345	13.1	0.000	5.75	0.005	7.87	0.004	6.54	0.003
DF ratio	− 1.69	0.130	− 1.59	0.156	− 1.58	0.153	− 1.38	0.239	− 0.21	0.849	1.32	0.257
Age	1.81	0.107	0.68	0.517			− 3.72	0.020^*^	− 3.23	0.048^*^		
Classification			− 0.17	0.869	1.62	0.144			− 2.46	0.091	− 2.83	0.047^*^
AICc	57.2	52.8	56.3	37.0	30.4	35.1						
BIC	56.6	52.0	55.7	34.4	25.4	32.5						
ROM, dorsiflexion												
	4.22	0.003	2.97	0.021	10.4	0.000	7.64	0.002	8.09	0.004	12.2	0.000
DF ratio	0.62	0.555	0.76	0.470	0.56	0.591	1.37	0.243	2.10	0.126	3.37	0.028^*^
Age	− 0.52	0.616	− 1.49	0.180			− 2.88	0.045^*^	− 1.87	0.159		
Classification			1.39	0.208	− 0.09	0.927			− 1.45	0.243	− 2.38	0.076
AICc	60.9	54.3	59.7	30.2	27.0	27.7						
BIC	60.3	53.5	59.1	27.6	22.1	25.1						
ROM, radial deviation												
	1.65	0.138	2.44	0.045	3.77	0.005	0.88	0.431	0.63	0.574	3.76	0.020
DF ratio	0.42	0.683	0.64	0.544	0.38	0.717	0.61	0.574	1.05	0.369	0.53	0.622
Age	− 0.30	0.773	− 1.88	0.102			0.57	0.597	1.01	0.389		
Classification			1.86	0.105	0.21	0.838			− 0.95	0.411	− 0.47	0.665
AICc	60.0	52.4	58.6	31.0	28.4	27.6						
BIC	59.4	51.6	58.0	28.4	23.5	25.0						
ROM, ulnar deviation												
	3.29	0.011	4.74	0.002	5.98	0.000	6.95	0.002	6.16	0.009	5.24	0.006
DF ratio	0.16	0.878	0.49	0.637	0.06	0.950	2.70	0.054	2.44	0.093	3.33	0.029^*^
Age	− 1.16	0.278	− 3.53	0.010^**^			− 4.19	0.014^*^	− 2.84	0.066		
Classification			3.16	0.016^*^	− 0.42	0.687			− 0.70	0.537	− 1.68	0.169
AICc	62.2	50.9	61.8	27.5	26.1	28.6						
BIC	61.6	50.1	61.2	24.9	21.2	26.0						
Arm circumference												
	7.26	0.000	1.72	0.129	23.3	0.000	6.89	0.002	7.73	0.005	14.9	0.000
DF ratio	− 3.01	0.017^*^	− 3.18	0.016^*^	− 2.62	0.031^*^	0.29	0.788	1.28	0.290	− 0.45	0.677
Age	2.31	0.050^*^	1.85	0.106			1.97	0.120	2.92	0.062		
Classification			− 1.19	0.274	1.66	0.136			− 1.68	0.191	-0.03	0.980
AICc	32.1	29.6	32.4	18.5	17.8	17.8						
BIC	31.5	28.7	31.8	15.9	12.9	15.1						
Forearm circumference												
	11.9	0.000	3.67	0.008	36.1	0.000	10.5	0.000	10.1	0.002	23.0	0.000
DF ratio	− 5.86	0.000^**^	− 5.73	0.001^**^	− 4.83	0.001^**^	2.63	0.058	1.53	0.224	0.39	0.715
Age	4.01	0.004^**^	1.90	0.100			2.87	0.046^*^	1.80	0.170		
Classification			− 0.75	0.480	3.02	0.016^*^			0.89	0.439	1.85	0.138
AICc	21.5	21.0	22.8	13.4	15.2	11.9						
BIC	20.9	20.2	22.2	10.8	10.3	9.28						

*DRUJ* Distal radioulnar joint, *GLMM* Generalized linear mixed model, *DF* Ratio; displacement-to-force ratio, *AICc* Corrected Akaike’s information criterion, *BIC* Bayesian information criterion, *ROM* Range of motion

*p < 0.05, ** *p* < 0.01

## Discussion

In some of the female wheelchair basketball athletes in this study, MRI findings showed the presence of TFCC injuries, and the ulnar deviation ROM was lower in the TFCC-injured wrists than in the intact wrists. In the intact wrists, DRUJ stability correlated with the forearm and upper arm strength.

In our study, MRI findings confirmed the presence of TFCC injuries in the wrists of 38.9% of female wheelchair basketball athletes. These athletes have been reported to have a high incidence of wrist injuries [3]. In a survey of mostly male non-elite wheelchair basketball athletes, 54.5% of respondents reported having injured their wrists in the past year [20]. This indicates the importance of preventing TFCC injuries in wheelchair basketball players of both sexes in elite or non-elite athletes. TFCC injuries cause pain in the ulnar side and sensations that the hand is being pulled [5, 21]. Wrist injuries affect the daily lives of wheelchair users, who use their upper limbs frequently and not just to control their wheelchairs. Furthermore, for wheelchair basketball athletes who require a high degree of upper limb performance during competition, preventing TFCC injuries is important not only for their daily lives but also for their high level of performance in competition.

There was no difference in DRUJ instability between the group with MRI findings of TFCC injuries and the group without, possibly due to the small sample size and the fact that MRI was performed after an average of 2.5 years since the TFCC injuries occurred. Individual differences in DRUJ instability have been reported, with different trends based on age and sex [22]. However, it was also reported that increased instability after TFCC injuries based on this assessment index is moderately predictable using the affected-to-unaffected ratio in patients with unilateral injuries [15]. Wheelchair users such as the participants of this study tend to use both arms in situations, including operating their wheelchairs or falling during competitions, which may be why the proportion of cases with bilateral injuries was high. Therefore, it may have been difficult to determine the severity of bilateral TFCC injuries using only the indicator of DRUJ instability calculated using this method. Furthermore, among patients with wrists showing MRI findings of TFCC, some presented with clinical symptoms. As other factors compensate for TFCC injuries, the presence or absence of clinical symptoms, such as DRUJ instability, may not always coincide with MRI findings. It is necessary to examine whether the presence of clinical symptoms in patients with MRI findings of TFCC injuries is related to DRUJ instability in future studies with more cases.

Wrists with MRI findings of TFCC injuries had more restricted ulnar deviation ROM than those without. As rest and protection using a supporter are a primary treatment for TFCC injury, it is possible that the ROM may have become restricted during the course following a TFCC injury. Additionally, as ulnar side pain can be caused by loading in the ulnar deviation position, such as in the TFCC load test, a limited ROM may have resulted from avoiding stress or positions that induce pain [7].

For the correlations in wrists with no MRI findings of TFCC injuries, higher grip strength and upper arm/forearm circumference were associated with a smaller displacement-to-force ratio (greater stability). In addition to TFCC, the extensor carpi ulnaris and pronator quadratus are also involved in DRUJ stability in the external support mechanisms [23–25]. With high grip strength and large forearm circumference, which are indicators of forearm muscle strength, the DRUJ external support mechanisms work properly and may contribute to DRUJ stability. However, we could not investigate the causal relationship between DRUJ instability and TFCC injuries because this was a cross-sectional survey of an athlete population at a single time point. As wrists with no MRI findings of TFCC injuries did not exhibit any injuries or symptoms at the time of the evaluations, it is necessary to investigate longitudinally whether maintaining or increasing grip strength and upper arm/forearm circumference is associated with preventing TFCC injuries in wheelchair basketball athletes. Further evaluation of the effects on daily life remains warranted, along with an assessment of the hand functions [26, 27].

This study has some limitations. First, owing to the limited number of elite athletes, the number of wrist joints measured in each group did not meet the minimal sample size determined using power analysis. As the overall proportion of elite athletes is low, multicenter or multinational studies are needed. Second, there is no evidence of an association between TFCC injury risk and DRUJ instability; therefore, the correlations between DRUJ instability and hand functions in intact wrists should be interpreted with care. Future longitudinal studies on the effectiveness of interventions to prevent TFCC injuries should be conducted to determine how best to prevent TFCC injuries and their recurrence in wheelchair basketball athletes.

## Conclusions

Female wheelchair basketball athletes with MRI findings of TFCC injuries exhibited a limited ulnar deviation ROM. In intact wrists, correlations were observed between hand functions such as upper arm/forearm strength and DRUJ stability evaluated using an ultrasound device. Longitudinal studies are needed to determine whether maintaining or increasing grip strength and upper arm/forearm circumference is a practical and effective intervention for preventing TFCC injuries in wheelchair basketball athletes. The results of this study could aid TFCC injury prevention and performance enhancement in athletes and could be of interest to coaches and medical professionals involved.

## Data Availability

The datasets generated and/or analyzed during the current study are not publicly available owing to ethical restrictions but are available from the corresponding author on reasonable request.

## Abbreviations

AICc: Corrected Akaike’s information criterion
BIC: Bayesian information criterion
DASH: Disability of the arm: shoulder: and hand
DRUJ: Distal radioulnar joint
GLMM: Generalized linear mixed model
MRI: Magnetic resonance imaging
ROM: Range of motion
TFCC: Triangular fibrocartilage complex

## References

[1] Cavedon V, Zancanaro C, Milanese C (2018). Anthropometry, body composition, and performance in sport-specific field test in female wheelchair basketball players. Front Physiol.

[2] Sakai M, Mutsuzaki H, Shimizu Y, Okamoto Y, Yatabe K, Muraki I (2021). Characteristic MRI findings of shoulder, elbow, and wrist joints in wheelchair user. Skeletal Radiol.

[3] Curtis KA, Black K (1999). Shoulder pain in female wheelchair basketball players. J Orthop Sports Phys Ther.

[4] Sakai M, Mutsuzaki H, Shimizu Y, Okamoto Y, Nakajima T (2022). Characteristic MRI findings of the shoulder, elbow, and wrist joints in elite wheelchair basketball players. BMC Sports Sci Med Rehabil.

[5] Andersson JK, Axelsson P, Stromberg J, Karlsson J, Friden J (2016). Patients with triangular fibrocartilage complex injuries and distal radioulnar joint instability have reduced rotational torque in the forearm. J Hand Surg Eur.

[6] Wijffels M, Brink P, Schipper I (2012). Clinical and non-clinical aspects of distal radioulnar joint instability. Open Orthop J.

[7] Skirven TM, Osterman AL, Fedorczyk JM, Amadio PC (2011). Rehabilitation of the hand and upper extremity.

[8] Hess F, Farshad M, Sutter R, Nagy L, Schweizer A (2012). A novel technique for detecting instability of the distal radioulnar joint in complete triangular fibrocartilage complex lesions. J Wrist Surg.

[9] Hess F, Sutter R, Nagy L, Schweizer A (2016). Stability and clinical outcome after reconstruction of complete triangular fibrocartilage disruption. J Wrist Surg.

[10] Oldfield CE, Boland MR, Greybe D, Hing W (2017). Ultrasound imaging of the distal radioulnar joint: a new method to assess ulnar radial translation in forearm rotation. J Hand Surg Eur.

[11] Yoshii Y, Yuine H, Tung WL, Ishii T (2019). Quantitative assessment of distal radioulnar joint stability with pressure-monitor ultrasonography. J Orthop Surg Res.

[12] International. IWBF player classification manual version 202110–1. https://iwbf.org/wp-content/uploads/2021/11/2021-IWBF-Classification-Manual-Version-202110-1.pdf, 2021; Accessed 2 Oct 2022

[13] Yoshii Y, Ishii T, Tung WL (2015). Ultrasound assessment of the effectiveness of carpal tunnel release on median nerve deformation. J Orthop Res.

[14] Yuine H, Yoshii Y, Tung WL, Ishii T, Shiraishi H (2019). Reliability of quantitative assessment of distal radioulnar joint stability with force-monitor ultrasonography. J Orthop Res.

[15] Yuine H, Yoshii Y, Iwai K, Ishii T, Shiraishi H (2022). Application of force-monitor ultrasonography to assess distal radioulnar joint instability in patients with triangular fibrocartilage complex injury. Ultrasound.

[16] Imaeda T, Toh S, Wada T, Uchiyama S, Okinaga S, Kusunose K (2006). Validation of the Japanese society for surgery of the hand version of the quick disability of the arm, shoulder, and hand (QuickDASH-JSSH) questionnaire. J Orthop Sci.

[17] Moriya T, Aoki M, Iba K, Ozasa Y, Wada T, Yamashita T (2009). Effect of triangular ligament tears on distal radioulnar joint instability and evaluation of three clinical tests: a biomechanical study. J Hand Surg Eur.

[18] Onishi T, Omokawa S, Iida A, Nakanishi Y, Kira T, Moritomo H (2017). Biomechanical study of distal radioulnar joint ballottement test. J Orthop Res.

[19] Cohen J (1992). A power primer. Psychol Bull.

[20] Soo Hoo JA, Latzka E, Harrast MA (2018). A descriptive study of self-reported injury in non-elite adaptive athletes. Pm r.

[21] Nakamura T, Berger RA, Fujita M, An K-N (2001). Distal radioulnar joint instability during forearm rotation: effect of muscle loading. J Jpn Soc Surg Hand.

[22] Yuine H, Yoshii Y, Iwai K, Ishii T, Shiraishi H (2021). Assessment of distal radioulnar joint stability in healthy subjects: changes with dominant hand, sex, and age. J Orthop Res.

[23] King GJ, McMurtry RY, Rubenstein JD, Ogston NG (1986). Computerized tomography of the distal radioulnar joint: correlation with ligamentous pathology in a cadaveric model. J Hand Surg Am.

[24] Johnson RK, Shrewsbury MM (1976). The pronator quadratus in motions and in stabilization of the radius and ulna at the distal radioulnar joint. J Hand Surg Am.

[25] Stuart PR (1996). Pronator quadratus revisited. J Hand Surg Br.

[26] Białkowska J, Juśkiewicz-Swaczyna B, Andrzejczak M (2021). Using the Jebsen-Taylor test in patients after radial bone fracture. Adv Rehab.

[27] Feitz R, van der Oest MJW, van der Heijden EPA, Slijper HP, Selles RW, Hovius SER (2021). Patient-reported outcomes and function after reinsertion of the triangular fibrocartilage complex by open surgery. Bone Joint J..

